# Development and characterization of carbon-based conductive pastes with high mechanical integrity under bending stress for room-temperature printable electronics

**DOI:** 10.1038/s41598-025-90210-0

**Published:** 2025-02-21

**Authors:** Santiago Mesa, Edwin Ramírez, Kelly G. Rivera Botia, Franklin Jaramillo, Daniel Ramírez

**Affiliations:** Centro de Investigación, Innovación y Desarrollo de Materiales – CIDEMAT, Facultad de Ingeniería, Universidad de Antioquia UdeA, Calle 67 No. 52-21, Medellín, 050010 Colombia

**Keywords:** Graphite, Carbon black, Carbon pastes, Printable electronics, Flexible electrodes, Bending test, Materials for devices, Materials for energy and catalysis

## Abstract

Room temperature processing of flexible electronics has become of great interest, as it allows for simpler and cheaper methodologies for high throughput manufacturing of printed electronics. This study focuses on the development and characterization of carbon-based conductive pastes made from a combination of graphite (G) and carbon black (CB), in a polymethyl methacrylate (PMMA) polymer matrix. Raw materials were characterized by Raman Spectroscopy, FTIR, SEM and TEM, showing the structural properties, morphologies and particles size which influenced the characteristics of the pastes. By varying the ratios of G/CB (1 to 4), carbon filler content (11.6–20%), and polymer content (1.5–7%), 48 different formulations were fabricated and further analyzed to determine their electrical conductivity as films. This process identified the optimal formulation for each G/CB ratio. Pastes with higher relative graphite content (G/CB ratios of 3 and 4) yielded the lowest resistivities (as low as 0.078 Ω cm) attributed to the effective formation of conductive networks between G and CB. Best-performing pastes were further characterized by sheet resistance, viscosity, adhesion, and scanning electron microscopy (SEM) analysis to understand the microstructure of the films. Flexible electrodes fabricated on PET substrates withstood 6000 bending cycles, thermal stress at 70 °C, and immersion in water, maintaining electrical conductivity. These results have significant implications for the future development of carbon-based conductive materials for room-temperature applications in flexible and printed electronics.

## Introduction

Printed and flexible electronics have attracted considerable attention due to their potential relevance in the field of photodetectors^1^, electrocatalysis^2^, energy harvesting^3^ and biosensing^4^. In this context, carbon-based pastes have emerged in a wide array of technological applications, ranging from conductive inks in printed electronics^5^ in supercapacitors^6^, electrode materials for third generation solar cells compatible with printing methods^7^, energy storage in batteries^8^, molecule sensing^9^ and electrodes for oxygen evolution reaction in water splitting^10^. In addition, carbon pastes are designed to enable fine-tune of both electrical and mechanical properties. One key approach to achieving this fine-tuning involves the careful manipulation of the composition and processing parameters, particularly the ratios of different carbon fillers and the polymer matrix that binds them^11^.

In this scenario, carbonaceous materials such as graphite and carbon black present advantages compared to metal particles, like relatively inertness in their final state^12^, cheaper and more abundant^13,14^, and excellent chemical stability^15^. Additionally, carbon pastes can be easily processed using solution-based techniques like screen printing^16^, blade coating and slot-die^17^.

On the other hand, PMMA (polymethyl methacrylate), is a stable, non-toxic material, safe for food and medical applications, with no significant direct environmental risks during use^18^. Its production involves the use of monomers such as methyl methacrylate (MMA), which requires proper handling. However, PMMA offers good weather resistance, water resistance^19^ and offers the possibility for room-temperature polymerization.

In this regard, for many carbon electrode applications, near-room-temperature processing is increasingly important. This approach enables more energy-efficient fabrication while preserving key properties such as low porosity, high electrical conductivity, and surface chemistry. Additionally, it ensures compatibility with a wide range of substrates and functional materials, including polymers, catalysts, and hybrid materials, which may not tolerate high-temperature processing. In the pursuit of low-temperature processable carbon pastes, various studies have explored the influence of different types of carbon fillers, such as graphite and carbon black, on the electrical conductivity and mechanical properties of the resulting films^20,21^. Graphite, with its high crystallinity, offers excellent electrical conductivity, while carbon black, known for its high surface area, facilitates the formation of conductive networks between particles^22,23^. However, the relative proportions of these fillers, along with the choice and amount of polymer, can significantly influence the overall performance of the paste^24^.

This study investigates the development and characterization of carbon pastes with varying compositions of graphite (G) and carbon black (CB), aiming at optimizing the balance of the electrical conductivity. By systematically varying the G/CB ratio, the content of carbon fillers, and the polymer content, a comprehensive set of pastes was fabricated and analyzed. The resistivity of these pastes was thoroughly characterized to determine the optimal formulations for low-resistivity, and the viscosity and mechanical properties of the best formulations were established.

The results indicate that the G/CB ratio plays a critical role in determining the resistivity of the pastes, with higher graphite content generally leading to lower resistivity, particularly at ratios of 3 and 4. This is attributed to the enhanced formation of conductive networks at these ratios. However, the interplay between carbon filler content and polymer concentration is also critical, as it influences the ability of the paste to form a continuous conductive film. The rheological behavior of the best pastes further reveals how composition affects the processability of the material, with higher carbon black content leading to higher viscosities and more complex flow behaviors. Best-performing electrodes fabricated on PET substrates withstood 6000 bending cycles, thermal stress at 70 °C, and immersion in water. These results have significant implications for the future development of carbon-based conductive materials for room-temperature applications in flexible and printed electronics, providing valuable insights into the formulation of carbon pastes.

## Results and discussion

**Fig. 1:**
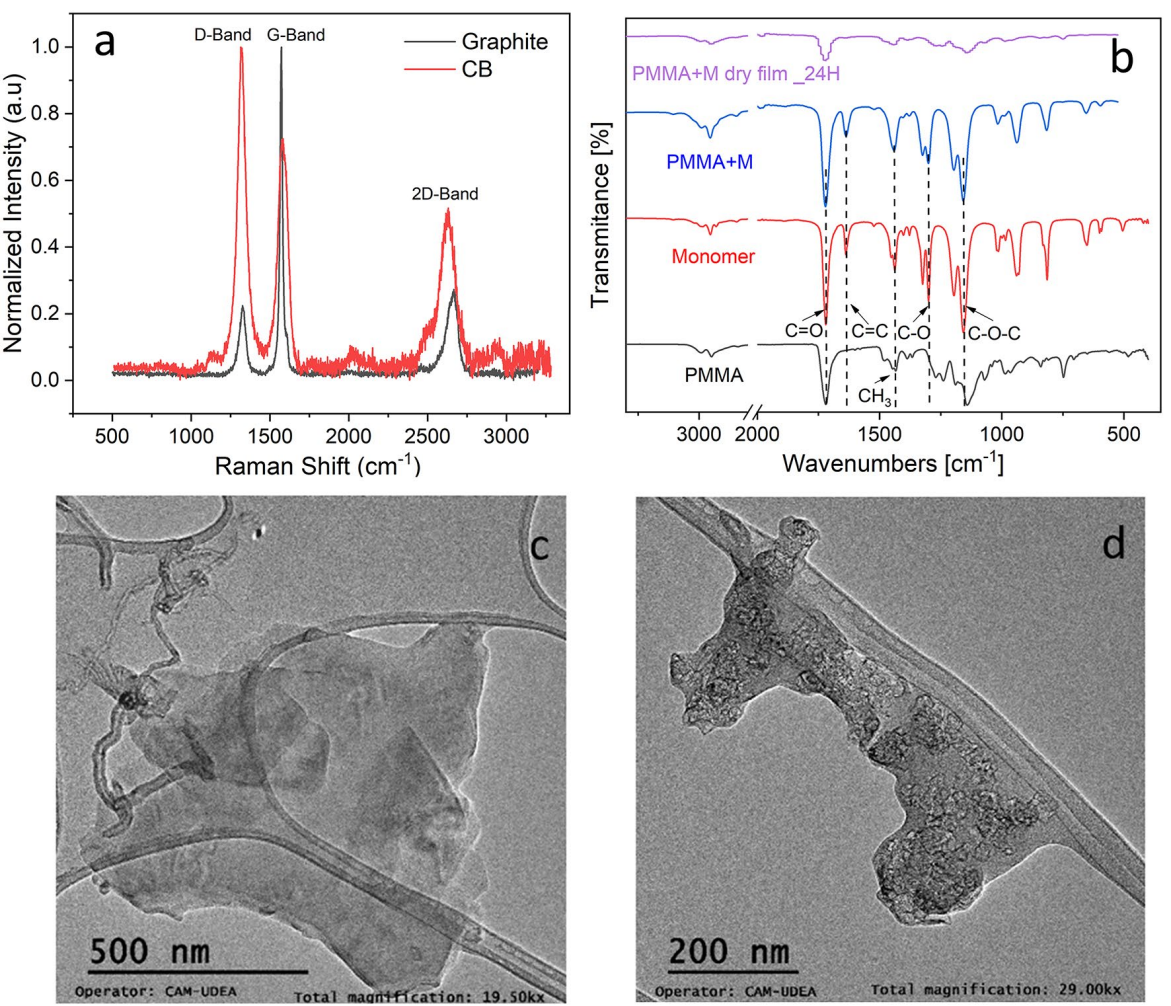
Characterization of raw materials by (**a**) Raman spectroscopy of the carbon materials, (**b**) FTIR spectra of the polymer and the formulated paste after 24 h drying, and Transmission electron microscopy (TEM) of (**c**) graphite, and (**d**) carbon black.

To obtain information about the crystalline defects in the materials, Raman spectroscopy was performed. Figure 1a presents the Raman spectra of the carbon materials used in the formulation of the conductive pastes. The bands observed at 1320 cm^−1^, 1580 cm^−1^, and 2630 cm^−1^ correspond to the D, G, and 2D bands, respectively^25,26^. The D band is typically associated with structural defects or disorders, including grain boundaries and edge defects. On the other hand, the G band is directly related to the crystallinity of the material, particularly to the vibrational mode of the carbon atoms in the hexagonal lattice. In contrast, the 2D band is related to purity, order, and the number of graphene layers in the structure. The lower the intensity of the 2D signal, the higher the purity and order of the graphene sheets. In this regard, the ratio of intensities of the D and G bands (I_D_/I_G_) is used as a measure of disorder of the carbon lattice, i.e., a higher ratio indicates more structural defects and worse electrical conductivity (non-graphitic carbon). Likewise, the intensity ratio of the G and 2D bands (I_G_/I_2D_) is associated with the number of graphene layers^15,27,28^.

In this context, Raman spectroscopy showed an I_D_/I_G_ intensity ratio of 0.224 for graphite, indicating a high degree of crystalline order. Likewise, carbon black presented an intensity ratio of 1.379, suggesting a greater number of structural defects. Also, the I_G_/I_2D_ intensity ratio was 3.663 for graphite, suggesting a higher number of graphene layers compared to carbon black which presents an intensity ratio of 1.402.

On the other hand, Fig. 1b shows the FTIR spectra for both, the prepolymer and the monomer used in the formulations. Two bands between 3000 cm^−1^ and 2900 cm^−1^ were present in the spectra, which corresponds to –CH sp^3^ stretching associated with carbon–hydrogen bonds. The band at 1722 cm^−1^, observed in both the PMMA and monomer spectra, is attributed to stretching of the C=O (*carbonyl*) bond, characteristic of polymers containing ester groups and related to the carbon–oxygen double bond^29–32^. At 1439 cm^−1^ and 1438 cm^−1^, peaks related to stretching of the C–H bonds in the polymer main chain are detected, particularly in the *methyl* (–CH_3_) groups of PMMA and monomer, respectively^29^. The peaks at 1141 cm^−1^ and 1158 cm^−1^ are characteristic of C–O–C (*ether*) bond stretching that is present in the ester structure of the polymer and monomer and is a typical signal of the vibration of C–O bonds^30^. In the monomer, the peak at 1637 cm^−1^ corresponds to the absorption of the *vinyl* group (C=C)^31^. This band is not observed in the infrared spectrum of PMMA, as it is a prepolymer with a high degree of polymerization. Finally, the band around 987 cm^−1^ is the characteristic absorption vibration of poly(MMA), and the two peaks at 1387 cm^−1^ and 753 cm^−1^, can be attributed to the vibrations of the *α-methyl* group^33^.

For the mixture of PMMA and monomer (PMMA + M), characteristic peaks corresponding to both PMMA and monomer were observed. The peaks at 2950 cm^−1^, 1730 cm^−1^ and 1150 cm^−1^ (C–H, C=O, C–O–C) indicate the presence of both, the polymer and the monomer in the sample. Once the paste was deposited and left to dry for 24 h (PMMA + M dry film), the decrease of the bands belonging to the monomer and polymer confirms the polymerization reaction. In addition, it is concluded that there is still a presence of components belonging to the PMMA and monomer, such as carbonyl groups (band 1722 cm^−1^) and C–H bonds (band around 1440 cm^−1^) and C–O–C (band 1140 cm^−1^).

Figure 1c and d show the morphology and size of graphite and carbon black, respectively. Graphite and carbon black had a size of 480 nm and 32 nm, respectively. As expected, graphite shows a flake-like morphology and carbon black a rounded particle one. Typically, these morphologies contribute to the interconnection between particles and create conductive pathways within the structure, as graphite flakes are covered with rounded carbon black nanoparticles^34,35^.

**Fig. 2:**
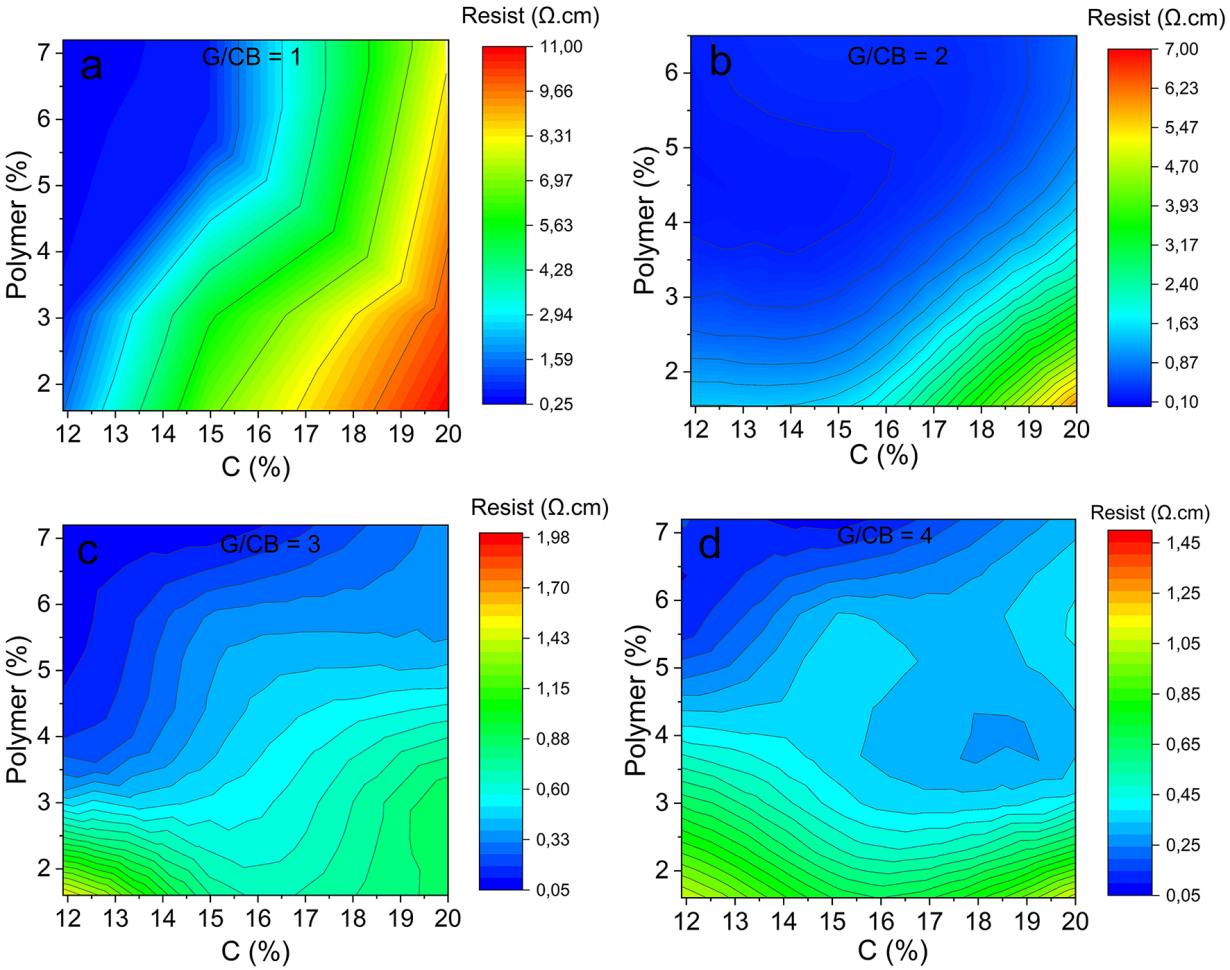
Change in resistivity at different C and Polymer percentages at: (**a**) G/CB = 1, (**b**) G/CB = 2 (**c**) G/CB = 3 and (**d**) G/CB = 4 ratios.

Different formulations of carbon pastes with variation of the G/CB ratios (1 to 4), carbon (G + CB) filler content (11.6–20%), and polymer content (1.5–7.2%) were fabricated and electrically characterized, as shown in Fig. 2a-d.

There is a clear trend where resistivity decreases as the G/CB ratio increases, especially when moving to ratios of 3 and 4. This result indicate that a higher relative graphite content significantly enhances conductivity within the paste, as already demonstrated in other works^15,36^. Accordingly, a more effective conductive network is established and highlights the positive influence of graphite content^37^. Additionally, as the G/CB ratio increases to 3 and 4, resistivity values drop across all percentages of carbon content. In comparison, at lower G/CB ratios resistivity is significantly higher, especially at higher percentages of carbon, suggesting that the conductive network is disrupted at these lower G/CB ratios due to the high relative content of carbon black. Although the carbon black establishes a conductive inter-particle network for the graphite, and partially fills interparticle voids^38^, the high surface area of the carbon black and its lower conductivity negatively influence the interaction between graphite particles. This results in large carbon black conduits which leads to a higher resistivity of the films^39^. From these results, it could be inferred that a film made solely with graphite would have lower resistivity. Nonetheless, it is known that it is necessary to have a balance between graphite and carbon black^40^. To corroborate this, a paste with solely graphite as a conductive agent was made and the obtained resistivity of the film was 1.515 ± 0.35 Ω cm, which is substantially higher than reference sample. So, this remarks the necessity to reach an optimized balance of G/CB ratio to achieve low resistivity of the pastes.

Figure 2 also shows the influence of the carbon and polymer content on resistivity. When carbon content is at its highest level (20%) resistivity values are higher across the whole range of tested polymer content, particularly for G/CB equal to 1 and 2. This indicates that polymer is insufficient to bond the particles network at that carbon load. For low and intermedium content of carbon (11.6 and 15%, respectively) the resistivity values decrease at high polymer content, highlighting the bonding character of the PMMA at these carbon content^41^. However, it is noticeable that increasing the carbon content does not necessarily lead to lower resistivities^42^. This behavior was observed for each one of the G/CB ratios tested denoting that at high carbon content, large carbon black conduits are promoted, hindering the connection between conductive graphite particles and resulting in a decrease in conductivity.

So, for this system carbon black acts as a conductive aid agent, facilitating the interconnection between graphite particles. However, to form effective conductive paths, a balance between polymer, graphite and carbon black content must be achieved^43^, as shown in Fig. 2.

Based on the results obtained, optimized formulations were successfully developed for each G/CB combination, as shown in Table 1. Although the resistivity values differ significantly from those of metallic electrodes containing copper or silver particles (10^−3^–10^−5^ Ω cm), they fall within the range reported for electrodes made with carbon paste (×10^−2^ Ω·cm), which typically require high-temperature drying processes (Table S1, Supporting Information).

**Table 1:** Optimized pastes for each G/CB ratio used.

G/CB	C (%)	R (Ω cm)
1	11.50	0.261 ± 0.018
2	11.50	0.121 ± 0.001
3	11.50	0.089 ± 0.006
4	11.50	0.078 ± 0.003

**Fig. 3:**
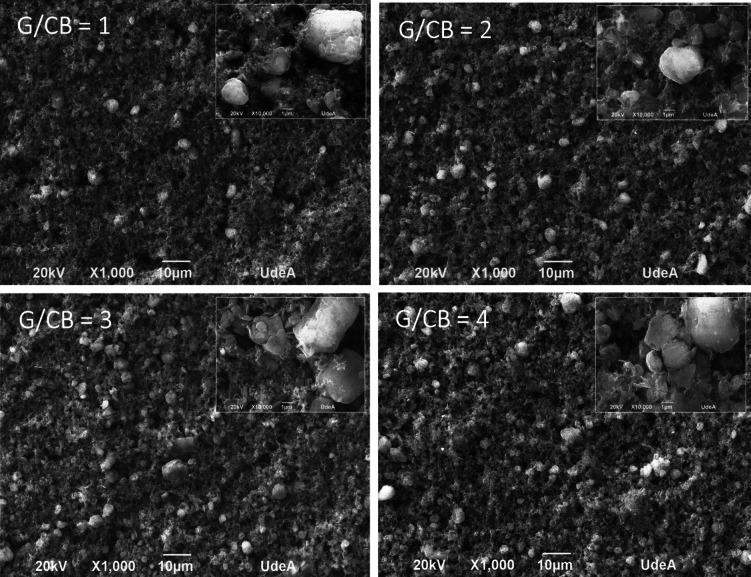
SEM images of the optimized compositions at each G/CB tested.

The films with the lowest resistivities obtained from each G/CB ratio were further morphologically characterized and tested. Figure 3 shows the SEM analyses for the optimized films of each G/CB. It was found that as the G/CB ratio increases, there is an agglomeration of graphite particles and less aggregates of carbon black particles in between, which favors the conductivity of the films. Also, at low G/CB ratios, larger agglomerations of carbon black particles were present resulting in larger conduction path between graphite particles, impairing the conductivity. These results agree with the results shown previously.

**Fig. 4:**
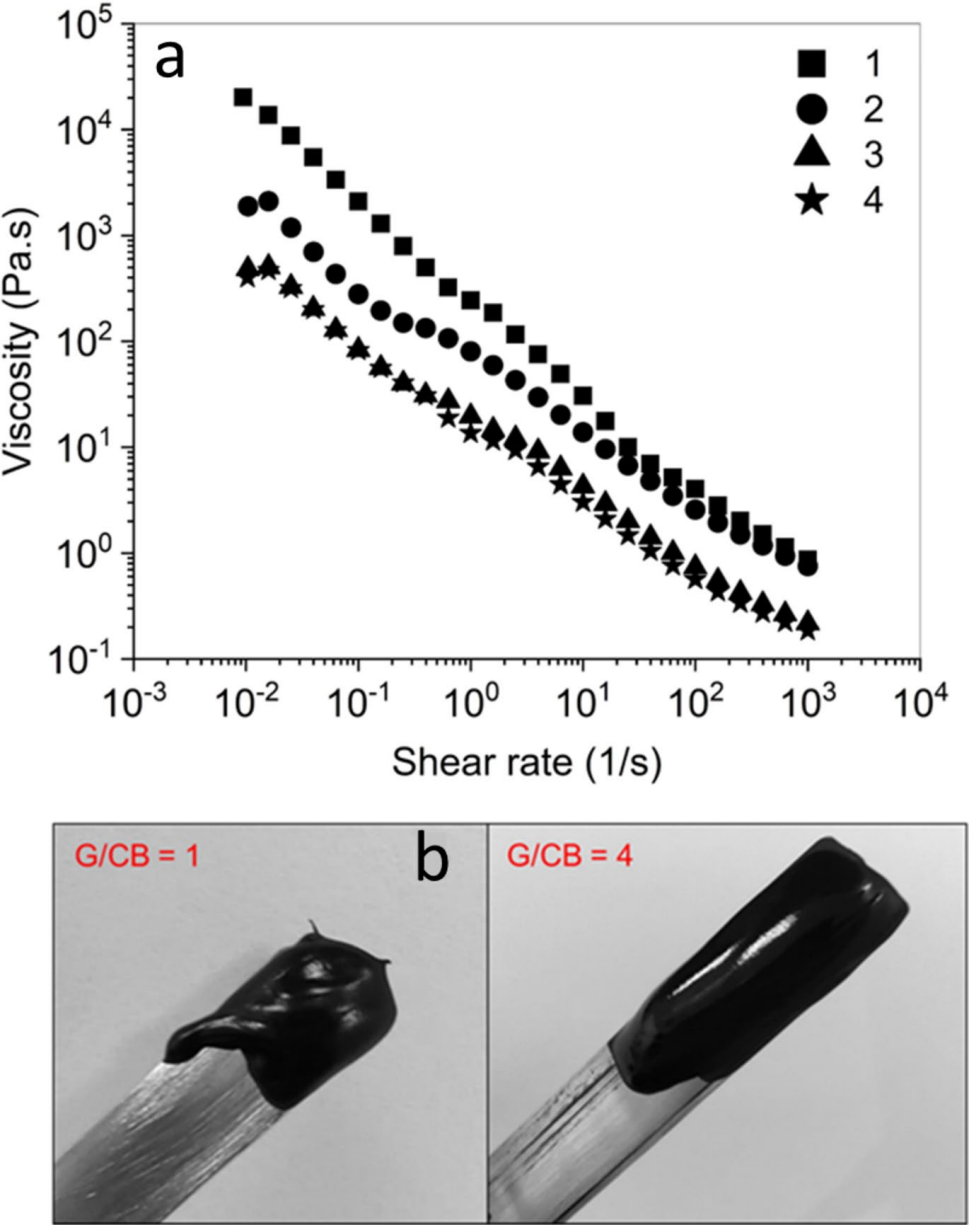
The flow curves of characteristics of Flow curves of (**a**) selected pastes and (**b**) physical appearance of the pastes.

As viscosity and flow properties of these pastes are of great relevance for adequate processing by different printing techniques, rheological characterization of these samples was also performed. Figure 4a shows the flow curve (i.e., viscosity as function of the shear rate) where all samples presented a non-Newtonian shear-thinning behavior across the different formulations. As the shear rate increases from 10^–2^ to 10^3^ s^−1^, the viscosity decreases significantly, indicating that the pastes become less viscous under higher shear forces which is a typical behavior of inks and pastes^44,45^. Particularly, higher viscosities were obtained for the formulations with lower G/CB ratios (1 and 2), which is due to the relatively high content of carbon black that makes the paste flow more difficult compared to the pastes with lubricant properties with higher graphite content^46^. This trend is also evident in the paste consistency by the comparison of G/CB ratios 1 and 4 in Fig. 4b. The lubricant effect of the graphite in nearly identical behavior for the 3 and 4 G/CB ratios across the entire shear rate range, whereas this effect was only observed for ratios 1 and 2 at high shear rates (above 10^1^ s^−1^). In this high shear rate region, the viscosity moved from 10^0^ to 10^1^ Pa s, which indicates that they are suitable for processing by blade coating and screen-printing techniques^47,48^. Viscosity also had a significant impact on the thickness of the carbon layers. Under identical Blade Coating fabrication conditions, film thicknesses ranging from 13 to 40 μm were achieved, with lower thicknesses observed for pastes with higher G/CB ratios (3 and 4) and higher thicknesses for those with lower G/CB ratios (1 and 2). This phenomenon can be attributed to the fact that, across all shear rate ranges, the viscosity of pastes with lower G/CB ratios (1 and 2) is greater, leading to a thicker wet and dry film. However, for the comparative analysis of electrical behavior, the resistivity was determined as the average of the product between sheet resistance and the film thickness.

To exploit the advantages of having room-temperature flexible electrodes, the best-performing pastes were also prepared by blade coating on PET substrates and their mechanical flexibility was evaluated by bending tests over thousands of bending cycles (2000–6000). SEM images (Fig. S1-4, supporting information) indicated that the layer with a G/CB ratio of 1 developed microcracks after drying, with the density and size of these cracks increasing as the number of cycles increased. Conversely, samples with G/CB ratios of 2, 3, and 4 did not show cracks in the as deposited dried films, but exhibited similar behavior, with cracks forming as the number of bending cycles increased. However, the cracks were smaller in size as the G/CB ratio increased.

**Fig. 5:**
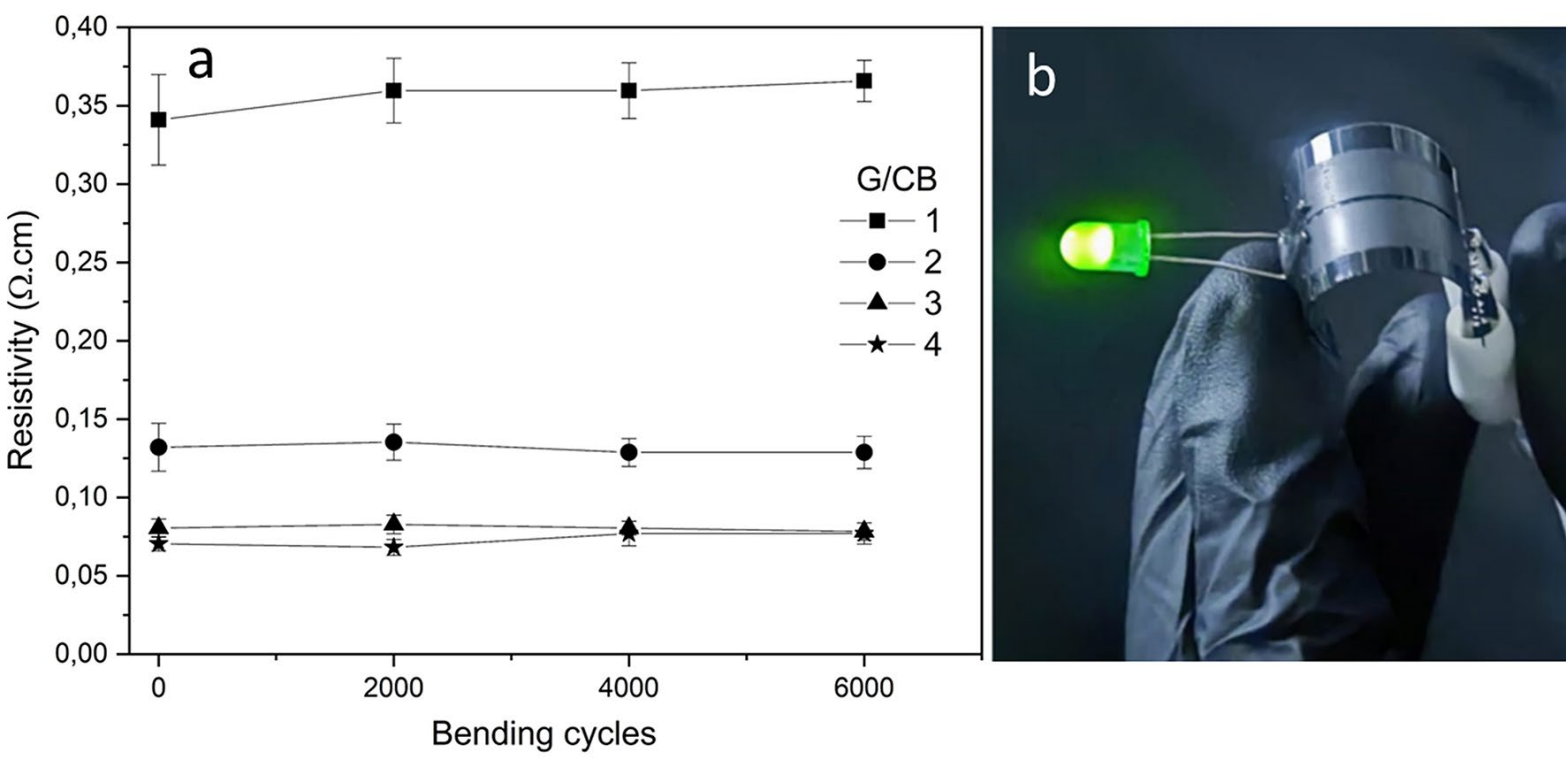
The change in resistivity at (**a**) different bending cycles and (**b**) demonstrated bent circuit using G/CB = 4.

Moreover, despite the presence of some cracks, the resistivity of the layers measured in the bent region showed no significant changes, as illustrated in Fig. 5a. This indicates that the fabricated layers maintain low resistivity under bending conditions, as shown in Fig. 5b. SEM images (Figures S1–S4) reveal that the cracks do not fully penetrate the cross-section of the layer, and the crack-free regions are sufficient to sustain electrical conduction without significant variation. This stability is attributed to the balance of the carbonaceous conductive material, even after 6000 bending cycles. The role of the carbonaceous filler in preserving electrical properties aligns with the behavior observed in carbon electrodes printed on textiles, where resistivity changes by only 5% after 2000 bending cycles^49^.

Additionally, the fabricated films showed thermal stability and water-proofing behavior for 96 h, remarking the mechanical robustness of the electrodes, as shown in Fig. S5.

To assess the adhesion of carbon layers of the different G/CB ratios, films were fabricated on flat glass and PET substrates (125 µm of thickness) using the blade coating technique. The adhesion test involved using an Elcometer cross hatch cutter to create a grid of incisions down to the substrate, followed by the application of 3 M Scotch® Magic™ tape. The portion of the film that detaches and adheres to the tape indicates the level of adhesion. According to ISO standards, the adhesion scale ranges from 0 to 5, with 5 indicating the lowest adhesion; in contrast, the ASTM scale ranges from 0 to 5B, with 0B representing the lowest adhesion.

**Fig. 6:**
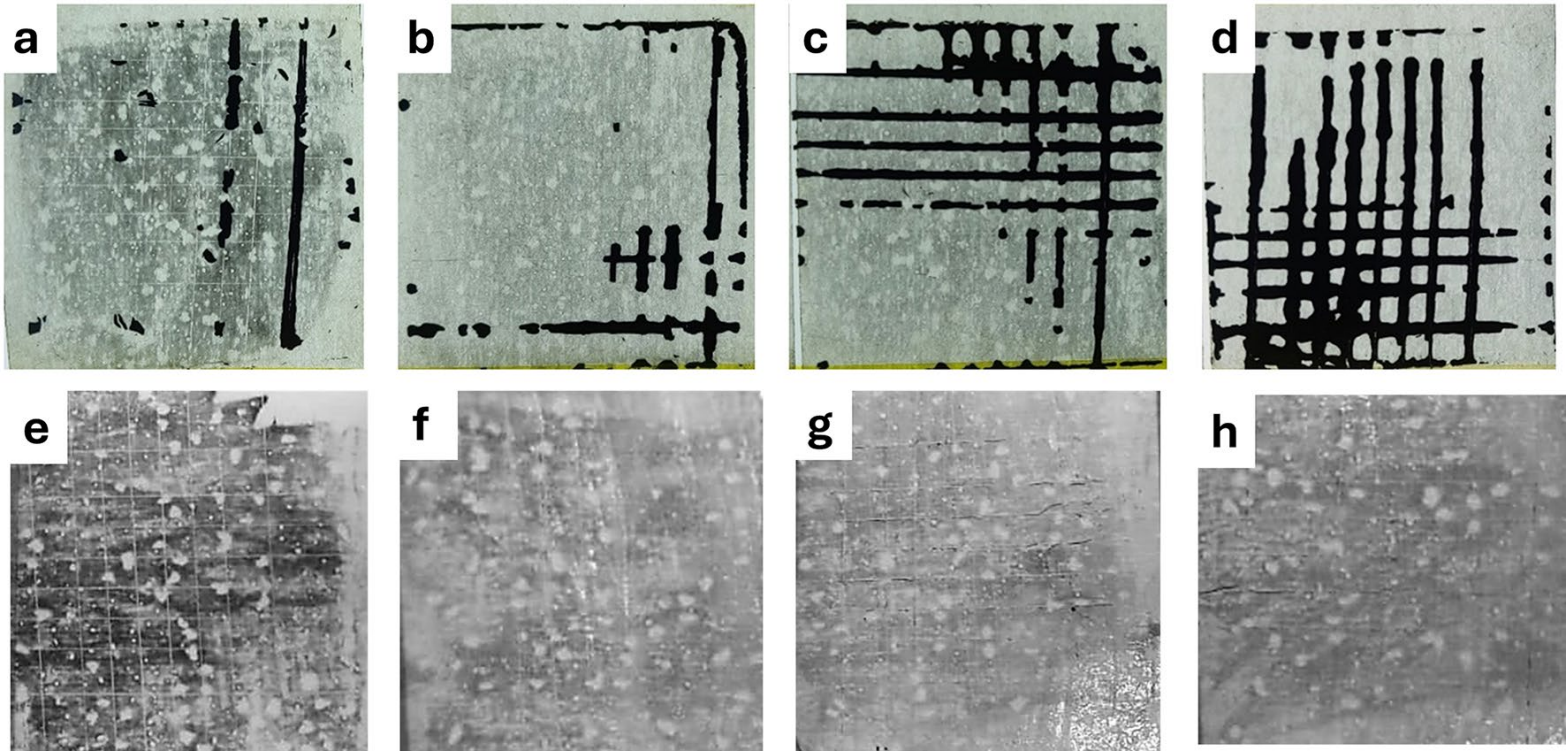
Images of the region transferred to the adhesive tape in the adhesion test for carbon electrodes on a glass substrate with G/CB ratios (**a**) 1, (**b**) 2, (**c**) 3 and (**d**) 4. Adhesion test for carbon electrodes on a PET substrate with G/CB ratios (**e**) 1, (**f**) 2, (**g**) 3 and (**h**) 4.

The film fabricated on a glass substrate with a G/CB ratio of 1 exhibited coating detachment at the intersections of the cuts, with a cross-sectional area not exceeding 5%, corresponding to a grade of 1 according to ISO standards and 4B under ASTM standards (Fig. 6). In contrast, the film with a G/CB ratio of 4 showed coating detachment at the cut intersections, covering a cross-sectional area between 15 and 35%, corresponding to a grade of 3 according to ISO standards and 2B under ASTM standards. These results indicate that the adhesion of the films improves significantly with an increased CB content. This enhancement is attributed to the smaller particle size of CB compared to graphite flakes, which provides a larger surface area for contact with the substrate, thereby improving adhesion. Furthermore, the adhesion test for the carbon film on a flexible substrate demonstrated superior adhesion (0 ISO and 5B ASTM) for all G/CB ratios, with only the G/CB ratio of 1 showing superficial carbon removal without substrate detachment.

## Materials and methods

### Materials

Carbon black (Alfa Aesar, ≥ 99.9%, acetylene, 50% compressed), graphite nanopowder (400 nm, 99.9%, NanoAmor), polymethyl methacrylate PMMA (> 99%, Mw = 800,000), Methyl methacrylate MMA (> 95%) were used for the fabrication of the pastes. The composition of the pastes varied by changing the G/CB ratio from 1 to 4. For each G/CB ratio two parameters were varied: content of carbon fillers from 11.6 to 20% and the content of PMMA from 1.5 to 7%. By doing so, 48 different pastes were obtained. All the components were mixed using a magnetic stirrer for at least 17 h.

### Characterization of raw materials

For the characterization of graphite and carbon black, RAMAN spectroscopy was performed using a Horiba Jobin Yvon confocal Raman spectrometer, Model Labram HR of high resolution, with focal length of 800 mm, laser spot size: from 1 to 300 mm, CCD detector with a resolution of 1024 × 256 pixels, optimized spectral range of 400–1100 nm, diffraction gratings of 1800 and 600 lines mm^−1^, spectral resolution of 0.3 cm^−1^ at 680 nm with 1800 lines/mm, spatial resolution of one lateral micrometer and two axial micrometers with 633 nm excitation. For the characterization of the polymerization reaction of the carbon paste, a ThermoScientific NicoletTM iS50TM Fourier Transform Infrared Spectrometer FTIR NicoletTM iS50TM was used using the ATR (attenuated total reflectance) method in a range between 550 cm^−1^ and 4000 cm^−1^ with a resolution of 4 cm^−1^; 4 samples were analyzed, named as PMMA, MMA monomer, the mixture of the two mentioned above (PMMA + M) and dry film after 24 h of PMMA + M deposition.

### Film processing and characterization

The resulting pastes were deposited using a blade coater machine (Elcometer 4340) using a speed of 3 mm s^−1^ and a gap of 175 µm. After the deposition no further treatment was done to the films. The sheet resistance (SR) of the obtained films was characterized using a four-point probe method, with co-linear electrical probes spaced 3 mm apart. The size of the samples for SR measurements was 2.5 × 2.5 cm. The thickness was measured by performing scratches in five areas of the carbon coating, spaced 5 mm apart. The depth of the scratches was determined using a Dektak XT profilometer (Bruker). The resistivity of the films was obtained by multiplying the SR of the film by their thickness. The films with the lowest resistivities obtained from each G/CB ratio were characterized by SEM (JEOL JSM 6490 LV), adhesion test and viscosity (TA instruments, Discovery HR-1).

The best films obtained were submitted to bending tests of 2000, 4000 and 6000 cycles at a radius consistency of 10 mm, speed of 5 mm/s, environmental conditions of 25° and relative humidity of 65%, and were further characterized by SEM and four-point probe method. For thermal test, the films were submitted for 96 h at 70 °C on a heat plate. Also, for waterproof test, the films were immersed in water for 96 h. For both thermal and waterproof tests, the SR was measured every 24 h. Finally, an adhesion test with 3 M Scotch® Magic™ tape and Elcometer Cross Hatch Cutter in accordance with the ASTM D3359 and UNE-EN ISO 2409:2021 standards was carried out on films fabricated on flat glass and PET substrates with a blade coater for each G/CB ratio.

## Supplementary Information

Supplementary Information.

## Data Availability

The data that support the findings of this study are available from the corresponding author, upon reasonable request.
