# Supplementary material for: Development and characterization of carbon-based conductive pastes with high mechanical integrity under bending stress for room-temperature printable electronics
Source: Sci Rep. 2025 Feb 21;15:6397. doi: 10.1038/s41598-025-90210-0 (PMC11845614; doi:10.1038/s41598-025-90210-0)
Supplement: Supplementary file 1 — Supplementary Information. [file 41598_2025_90210_MOESM1_ESM.docx]

**SUPPLEMENTARY INFORMATION**

**Development and characterization of Carbon-Based Conductive Pastes with High Mechanical Integrity Under Bending Stress for Room-Temperature Printable Electronics**

Santiago Mesa^1^, Edwin Ramírez^1^, Kelly G. Rivera Botia^1^, Franklin Jaramillo^1^, Daniel Ramírez^1^*

*estiben.ramirez@udea.edu.co

*^+^*Centro de Investigación, Innovación y Desarrollo de Materiales – CIDEMAT, Facultad de Ingeniería, Universidad de Antioquia UdeA, Calle 67 No. 52-21, Medellín, 050010 Colombia

| 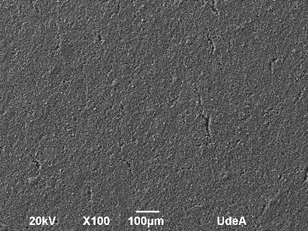  a | 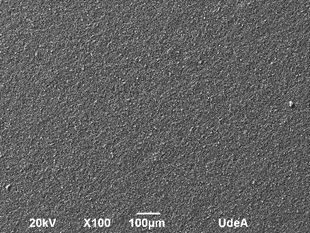  b |
| --- | --- |
| 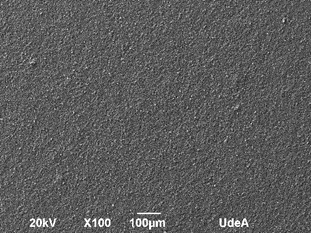  c | 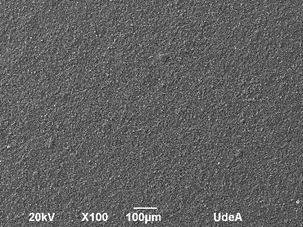  d |

**Fig S1** a) to d) SEM images of carbon electrodes before the bending test with G/CB ratios 1- 4, respectively.

| 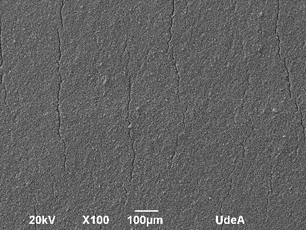  a | 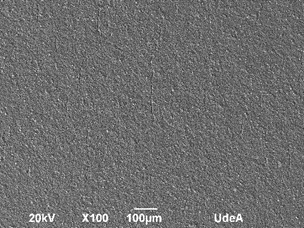  b |
| --- | --- |
| 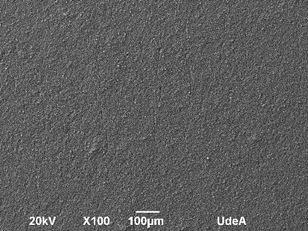  c | 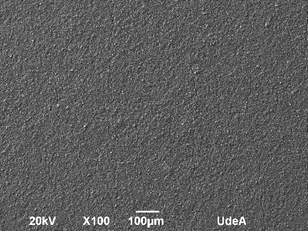  d |

**Fig S2** a) to d) SEM images of carbon electrodes after 2000 bending test cycles with G/CB ratios 1- 4, respectively.

| 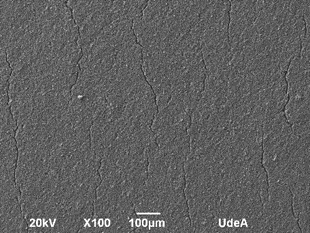  a | 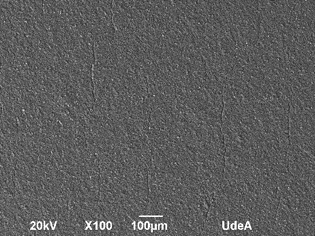  b |
| --- | --- |
| 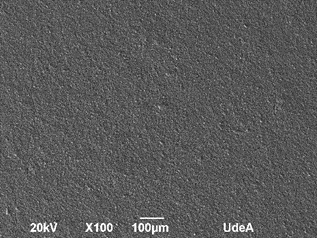  c | 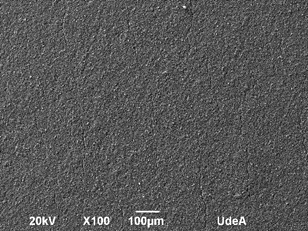  d |

**Fig S3** a) to d) SEM images of carbon electrodes after 4000 bending test cycles with G/CB ratios 1- 4, respectively.

| 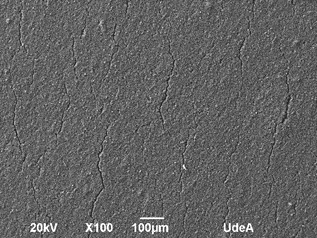  a | 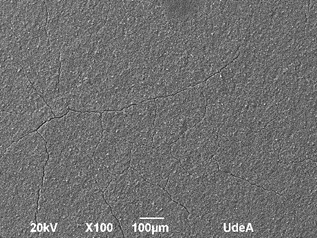  b |
| --- | --- |
| 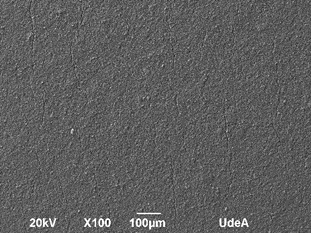  c | 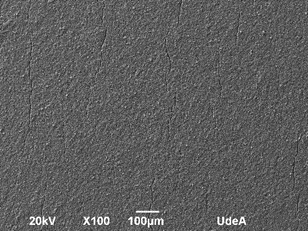  d |

**Fig S4** a) to d) SEM images of carbon electrodes after 6000 bending test cycles with G/CB ratios 1- 4, respectively.

| 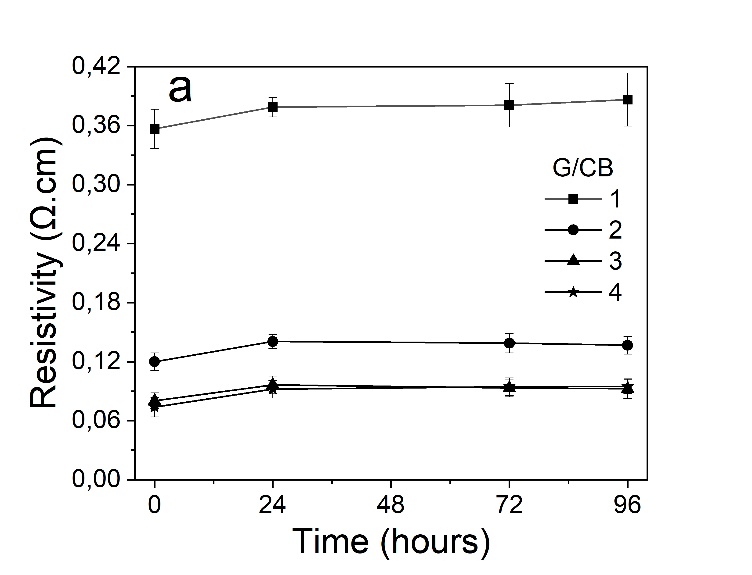 | 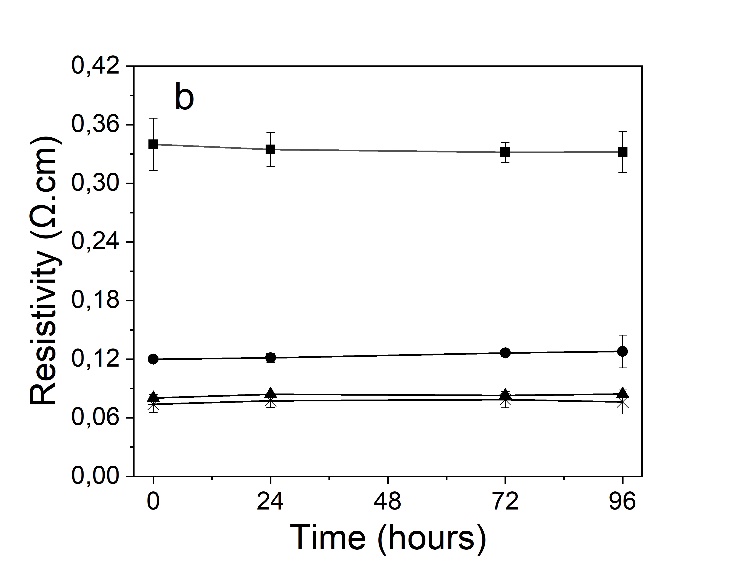 |
| --- | --- |

**Fig S5.** The change in resistivity of the best-performing carbon electrodes under **(a)** thermal stress at 70°C and **(b)** immersion in water, for 96 hours.

**Table S1.** Conductive paste formulations, composition and resistivity values

| **Electrode material** | **Binder** | **Curing Temperature** | **Resistivity (Ω.cm)** | **Fabrication methode** | **Ref** |
| --- | --- | --- | --- | --- | --- |
| Graphite/Carbon Black | Titanium (IV) isopropoxide | <100°C | 2.0x10^-2^ | Screen printing | [1] |
| Carbon nanoparticle (18 nm)/Graphite (5um) | Adhesive resin/ZrO2 | 70°C | 3.3x10^-2^ | Blade coating | [2] |
| Graphite/Carbon Black | PMMA | 100 °C | 3.3x10^-2^ | Blade coating | [3] |
| Carbon Black/Flaky graphite powder (1:0) | Polyvinyl acetate | 85°C | 4.4x10^-3^ | Hot pressing | [4] |
| Graphene nanoplatelet / Silver flakes | Not repoted | 250°C | 1.1x10^-3^ | Mesh stencil | [5] |
| silver microflakes/ silver microparticles | Epoxy resin | 120°C | 8.1x10^-3^ | Screen printed | [6] |
| Copper fillers | polypropylene carbonate | 200°C | 1.1x10^-3^ | Screen printing | [7] |
| Cu NPs and Cu flakes | Ethyl cellulose | 120 °C | 2.8 x10^-5^ | Screen printing | [8] |
| Cu NWs | Not reported | 140°C | 1.5 x10^-5^ | Mask printing | [9] |

**References:**

[1] P. Jiang, T.W. Jones, N.W. Duffy, K.F. Anderson, R. Bennett, M. Grigore, P.
Marvig, Y. Xiong, T. Liu, Y. Sheng, L. Hong, X. Hou, M. Duan, Y. Hu, Y. Rong, G.J.
Wilson, H. Han, Fully printable perovskite solar cells with highly-conductive, low-temperature, perovskite-compatible carbon electrode. Carbon 129 (2018) 830–836

[2] Zhou, H., Shi, Y., Wang, K., Dong, Q., Bai, X., Xing, Y., ... & Ma, T. (2015). Low-temperature processed and carbon-based ZnO/CH3NH3PbI3/C planar heterojunction perovskite solar cells. The Journal of Physical Chemistry C, 119(9), 4600-4605.

[3] Behrouznejad, F., Forouzandeh, M., Khosroshahi, R., Meraji, K., Badrabadi, M. N., Dehghani, M., ... & Taghavinia, N. (2020). Effective Carbon Composite Electrode for Low‐Cost Perovskite Solar Cell with Inorganic CuIn0. 75Ga0. 25S2 Hole Transport Material. Solar RRL, 4(5), 1900564.

[4] Wei, H., Xiao, J., Yang, Y., Lv, S., Shi, J., Xu, X., ... & Meng, Q. (2015). Free-standing flexible carbon electrode for highly efficient hole-conductor-free perovskite solar cells. Carbon, 93, 861-868.

[5] Ismail, I., Salim, M. A., Masripan, N. A., Saad, A. M., Akop, M. Z., Chew, K. W., & Photong, C. (2024). Resistivity of graphene/silver hybridization conductive ink on bending test. Journal of Advanced Research in Applied Mechanics.

[6] Maeda, K., Kashiwagi, Y., & Uno, M. (2021). Electrical and cyclic bending properties of screen-printed conductive patterns containing different ratios of silver microparticles and silver microflakes. Microelectronics Reliability, 123, 114243.

[7] Tang, J., Mak, C. H. H., Tam, S. K., & Ng, K. M. (2021). Formulation of a paste for copper thick film. Journal of Nanoparticle Research, 23(8), 166.

[8] Tam, S. K., Fung, K. Y., & Ng, K. M. (2016). Copper pastes using bimodal particles for flexible printed electronics. Journal of Materials Science, 51, 1914-1922.

[9] Zhang, B., Chen, C., Li, W., Yeom, J., & Suganuma, K. (2020). Well‐Controlled Decomposition of Copper Complex Inks Enabled by Metal Nanowire Networks for Highly Compact, Conductive, and Flexible Copper Films. Advanced Materials Interfaces, 7(1), 1901550.
